# Automated mesenchymal stem cell segmentation and machine learning-based phenotype classification using morphometric and textural analysis

**DOI:** 10.1117/1.JMI.8.1.014503

**Published:** 2021-02-01

**Authors:** Sakina M. Mota, Robert E. Rogers, Andrew W. Haskell, Eoin P. McNeill, Roland Kaunas, Carl A. Gregory, Maryellen L. Giger, Kristen C. Maitland

**Affiliations:** a Texas A&M University, Department of Biomedical Engineering, College Station, Texas, United States; b Texas A&M Health Science Center, College of Medicine, Bryan, Texas, United States; c University of Chicago, Department of Radiology, Committee on Medical Physics, Chicago, Illinois, United States

**Keywords:** image segmentation, cell phenotype classification, machine learning, stem cell, monolayer cell culture, viability assessment

## Abstract

**Purpose**: Mesenchymal stem cells (MSCs) have demonstrated clinically relevant therapeutic effects for treatment of trauma and chronic diseases. The proliferative potential, immunomodulatory characteristics, and multipotentiality of MSCs in monolayer culture is reflected by their morphological phenotype. Standard techniques to evaluate culture viability are subjective, destructive, or time-consuming. We present an image analysis approach to objectively determine morphological phenotype of MSCs for prediction of culture efficacy.

**Approach**: The algorithm was trained using phase-contrast micrographs acquired during the early and mid-logarithmic stages of MSC expansion. Cell regions are localized using edge detection, thresholding, and morphological operations, followed by cell marker identification using H-minima transform within each region to differentiate individual cells from cell clusters. Clusters are segmented using marker-controlled watershed to obtain single cells. Morphometric and textural features are extracted to classify cells based on phenotype using machine learning.

**Results**: Algorithm performance was validated using an independent test dataset of 186 MSCs in 36 culture images. Results show 88% sensitivity and 86% precision for overall cell detection and a mean Sorensen–Dice coefficient of 0.849±0.106 for segmentation per image. The algorithm exhibited an area under the curve of 0.816 (${\mathrm{CI}}_{95}=0.769$ to 0.886) and 0.787 (${\mathrm{CI}}_{95}=0.716$ to 0.851) for classifying MSCs according to their phenotype at early and mid-logarithmic expansion, respectively.

**Conclusions**: The proposed method shows potential to segment and classify low and moderately dense MSCs based on phenotype with high accuracy and robustness. It enables quantifiable and consistent morphology-based quality assessment for various culture protocols to facilitate cytotherapy development.

## 1 Introduction

Chronic diseases such as cancer, diabetes, stroke, obstructive pulmonary disease, renal failure, and arthritis are leading causes of disability and death. According to the National Center for Chronic Disease Prevention and Health Promotion, six in 10 adults in the US have a chronic disease and four in 10 adults in the US have two or more.^1^ The high prevalence of these diseases calls for effective treatments that can provide a long-term cure. Studies show that the approach of cell therapies has valuable potential to address this problem.^2^^–^^5^ Cell therapy is the transplantation of laboratory-expanded cells into patients to restore normal function by replacing damaged cells or by altering the physiology of the host in favorable ways. Mesenchymal stem cells (MSCs), a heterogeneous group of stem cells, have gained attention for clinical applications in regenerative medicine and tissue engineering ^6^^–^^10^ over the past few years. There is a growing body of literature demonstrating their therapeutic efficacy in a variety of pre-clinical models, including chronic renal failure,^11^ skeletal regeneration,^12^ and acute myocardial infarction.^13^

The success of cytotherapies to provide new remedies is highly reliant on the viability and reproducibility of cultured cell properties. In the past, cell quality has been commonly characterized and assessed with the help of their morphological characteristics.^14^^,^^15^ MSCs that rapidly self-replicate (RS) are spindle-shaped and fibroblastic, whereas cells that slowly replicate (SR) are flattened and rhomboidal.^16^ In addition to slow replication, SR cells lose most of their ability to differentiate into multiple cell lineages and promote tissue repair. Standard evaluation techniques such as labeling, flow cytometry, and *in vitro* assays are invasive and time-consuming making real-time culture monitoring impossible.^17^^–^^19^ Regular examination of cultures under a microscope is another qualitative approach that is used for routine quality assurance. However, visual inspection is highly subjective and tedious making it less reliable and robust. Therefore, a non-invasive and objective method is necessary to address these limitations.

Computer vision/artificial intelligence is a very promising method to quantitatively predict culture quality from images of MSCs based on their morphological phenotype. The topic of cell image analysis has received great recognition with the increasing demands in bioinformatics^20^ and significant contributions in the field of medical diagnostics and biomedical applications.^21^^,^^22^ Morphological cell image analysis has become a standard theory for computerized processing and pattern recognition and it encompasses a rather wide application area, such as cell clump segmentation, morphological feature extraction, and abnormal cell identification.^23^^–^^25^ It has also been integrated with the study of histological tumor sections,^26^ boundary detection of epithelial cell nuclei,^27^^,^^28^ or understanding drug influences.^29^ Image-based segmentation of MSCs reported previously^30^ demonstrated better results compared to conventional thresholding techniques. However, this work was more driven toward identifying all cell regions rather than individual cells, making it unsuitable for culture quality monitoring through morphological profiling of each cell. In addition to segmentation, there has been significant research demonstrating the possibility of characterizing MSCs based on their shape.^31^ Machine learning has been implemented previously to classify MSCs from other cell lines,^32^^,^^33^ to predict immunosuppressive capacity using their functional subpopulations,^34^ and also to identify them based on their differentiation potential.^35^ This idea could be similarly extended to differentiate MSCs depending on their efficacy as indicated by their morphological phenotypes (RS and SR).

Building on our preliminary work,^36^ the research reported here presents an integrated approach to segment and classify MSCs in phase micrographs, potentially providing automated analysis of monolayer culture viability. This is achieved by the development and evaluation of

• an algorithm to localize and segment individual MSCs and MSCs in clusters from images of low and moderate cell density, and
• a machine learning model using morphological and textural features extracted from segmented cells to distinguish between RS and SR phenotypes of MSCs.

The efficiency and performance of automated cell segmentation is strongly dependent on imaging modality. Fluorescence and phase-contrast microscopy are the two most widely applied techniques for acquisition of cell images with improved image contrast. Fluorescence microscopy is prone to photobleaching, which limits its applications in long-term monitoring.^37^ Also, in fluorescence imaging, cells are typically stained or genetically engineered to generate fluorescent proteins to enhance cell boundary information, which may cause changes to the cellular morphology and dynamics, potentially invalidating their use in humans.^38^ Thus, this imaging technique is less desirable for a non-invasive and real-time approach. This is overcome by phase-contrast microscopy, an optical imaging method that converts the phase shifts in light passing through a specimen into intensity changes in the image. It is based on the principle that the difference in the refractive index between the cells and the substrate causes phasing that provides relatively high image contrast in micrographs without any biological modification to cells.^39^ Phase-contrast microscopy is a standard technique for visual inspection and evaluation of MSC morphology.

Here, segmentation of MSCs is handled as a three-step approach, where it first localizes regions in the image that contain cells, then finds algorithm-defined markers, and finally integrates the regions with markers to segment individual cells inside clusters. The individual cells obtained with the algorithm can be analyzed further to draw conclusions about the culture population. In addition to segmenting MSCs, features that are potential indicators of the physiological state of the MSCs are also computed. These features are used by a machine learning model to classify the phenotype of each cell as RS or SR. Based on the phenotype of the cells from culture images, the algorithm will be able to provide the proportion of maximally efficacious cells in the culture. Thus, the developed image analysis protocol is novel in its contribution to automated and rapid image-based processing to objectively examine the efficacy of adherent MSCs cultures. Another innovative aspect of this work is the implementation of a comprehensive top-to-bottom computer vision pipeline to identify MSCs and predict their relationship to RS or SR morphological phenotypes. Moreover, its potential to replace or augment visual inspection would make cell culture evaluation rapid, quantitative, and less tedious, rendering it beneficial for scale-up of cell manufacturing. Beyond validation of the applicability of this image analysis algorithm for cell quality control, an advantage of this work is its promise for streamlining culture processes for cell therapy development and manufacturing.

## 2 Methods

### 2.1 Overview

**Fig. 1:**
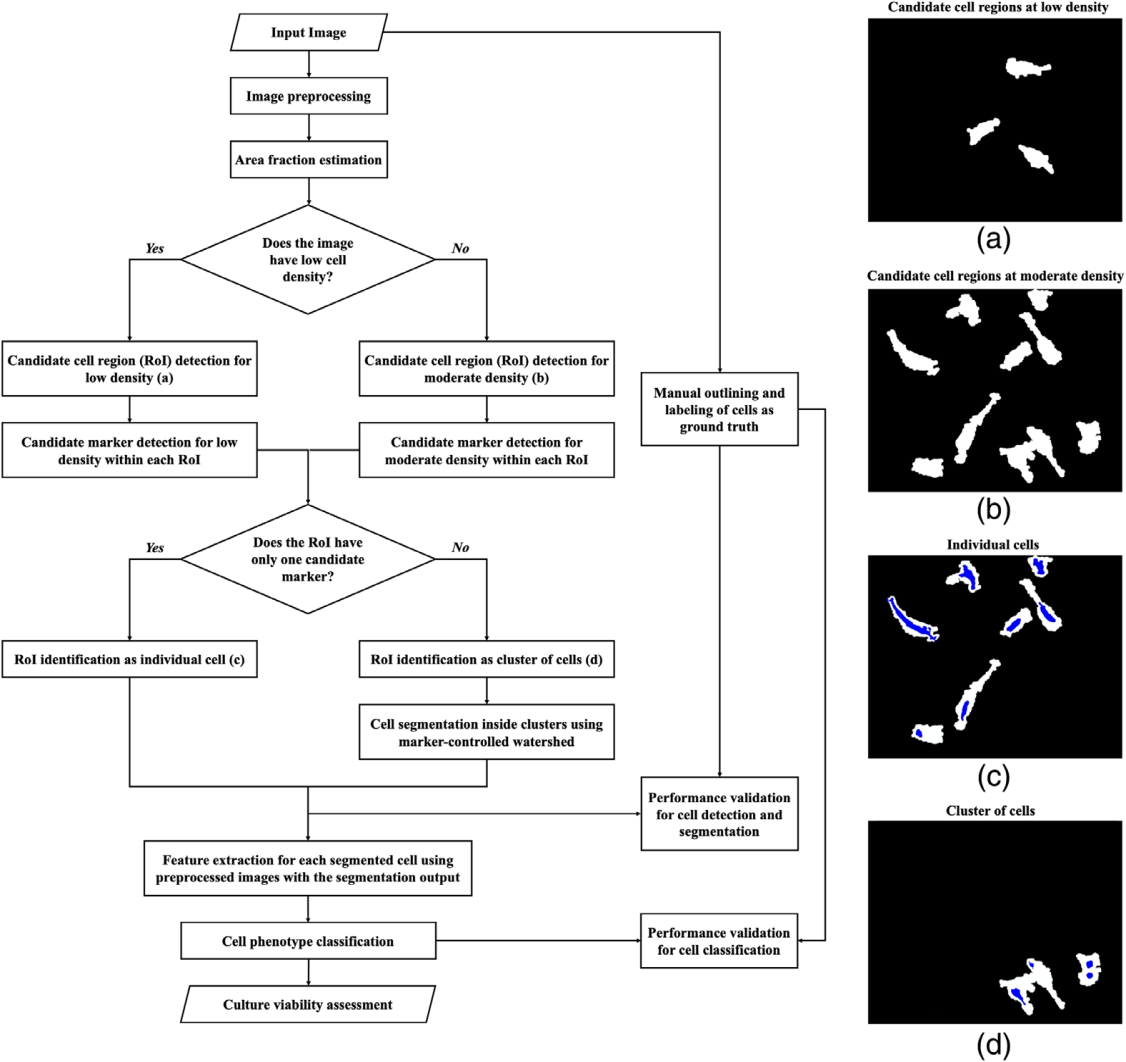
Pipeline of algorithm to classify mesenchymal stem cells in phase-contrast micrographs. Example images illustrate (a) low and (b) moderate density. Cell ROI in image (b) include (c) individual cells and (d) clusters of cells, differentiated by the number of candidate markers (blue) inside RoIs (white).

The image analysis approach was developed to classify MSC phenotype using phase-contrast micrographs of monolayer culture. Figure 1 shows the overall flow of the method; each step is detailed in the subsequent sections. Following preprocessing, the algorithm estimates cell density. Morphological operations and thresholding detect regions of the image that contain cells. Candidate markers are localized within these regions to identify if the cell region is a single cell or cell cluster. A cell cluster is further segmented to identify individual cells. After the segmentation of each cell, the algorithm extracts several human-engineered morphometric and textural features. Cell segmentation and feature extraction algorithms are built using the comprehensive set of reference-standard algorithms provided by the Image Processing Toolbox in MATLAB 9.5 (R2018b). Machine learning classifiers are trained using these features to distinguish between the RS and SR phenotype. Classification models were developed in Jupyter Notebook 6.0.1 using Python 3.5.6 libraries.

### 2.2 Dataset

Human bone marrow-derived MSCs were seeded at $100\;\text{cells}/{\mathrm{cm}}^{2}$ under standard conditions of expansion^40^ and imaged on the second and fourth day after the culture was prepared to capture variation in phenotype as cells proliferate. For the MSCs employed in this study, a density of $∼1000\;\text{cells}/{\mathrm{cm}}^{2}$ is expected at day 2 and $∼6500\;\mathrm{per}\;{\mathrm{cm}}^{2}$ at day 4.^12^ A Motic AE31 phase-contrast microscope with a 10× objective and Moticam 1SP 1.0 MP camera was used to acquire culture images. All the images collected for this study have a size of 1280×1024  pixels and a resolution of 1.56  pixels/μm. Cell culture and image capture were repeated three times to generate the dataset for training and testing of the algorithm. Using Adobe Photoshop and Microsoft Paint software, cells were manually segmented and labeled as RS or SR phenotype by an individual with more than 15 years of experience in culturing MSCs. Images from two cultures served as ground truth for training, and images from the third culture were used for independent testing as detailed in Table 1. The training dataset of 71 images consisted of 472 cells with 307 cells labeled as RS and 165 cells as SR. The algorithm was validated with 36 phase-contrast micrographs having 186 cells with 121 RS cells and 65 SR cells. Each cell from the segmented ground truth was also characterized as RS or SR by 20 people trained to visually identify MSC phenotype to further analyze the generalizability of the method.

**Table 1:** Mesenchymal stem cell culture dataset.

	Culture day	No. of images	No. of cells	No. of RS cells	No. of SR cells
Culture 1	2	15	76	49	27
(Train)	4	15	146	80	66
Culture 2	2	17	96	73	23
(Train)	4	24	154	105	49
Culture 3	2	15	64	46	18
(Test)	4	21	122	75	47

### 2.3 Image Preprocessing and Area Fraction Estimation

**Fig. 2:**
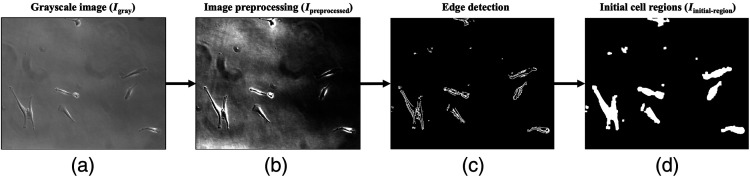
Example image of MSCs shows the steps involved in detecting initial cell regions. (a) The input phase-contrast micrograph is converted to grayscale and (b) preprocessed using contrast adjustment, sharpening, and anisotropic filtering. (c) Edges are detected using Sobel operator. (d) Edges are connected and filled using dilation, closing, and flood-fill operation to obtain initial cell regions ($I_{\text{initial}-\text{region}}$).

The input RGB phase-contrast micrograph is converted to grayscale ($I_{\text{gray}}$), shown in Fig. 2(a), and preprocessed to reduce the effect of undesired imperfections introduced during imaging. Contrast of $I_{\text{gray}}$ is adjusted to increase the intensity variation between the cells and the substrate, making cell regions more detectable for segmentation. Cell edges are sharpened using unsharp masking and then filtered using anisotropic median-diffusion to remove unwanted artifacts while improving the signal-to-background ratio without distorting edges [Fig. 2(b)].^27^ The preprocessed image ($I_{\text{preprocessed}}$) is further processed by Sobel filter to identify cell boundaries in the images [Fig. 2(c)]. Sobel operator highlights regions with maximum intensity change, detecting edges above a sensitivity threshold of 1. Once object outlines are obtained, dilation and closing are performed to connect the detected edges. This is followed by flood-fill operation to remove holes from the filled regions.

Using the same structuring element sizes for morphological operations to process images with a high number of cells and images with a low number of cells leads to poor segmentation performance. Apart from the number of cells and clusters, the variances in their size and shape also become significant as the culture grows over time. Using area fraction (AF) as a deciding factor for cell density addresses this problem as different parameters are used for low and moderate density levels to identify cell regions and the markers inside them. Also, every image is evaluated based on their density estimate rather than through the potentially erroneous assumption that duration in culture is a robust predictor of density. The cell density-based criterion automatically triggers optimizations in the algorithm so as to ensure comparable performance at a range of cell density levels.

AF of binary image ($I_{\text{initial}-\text{region}}$) is used to obtain an estimate of the input micrograph’s cell density. It is calculated by the algorithm as the percentage of white pixels in the image as given as

$$\mathrm{AF}=\frac{\overset{M}{\underset{x=1}{\sum }}\overset{N}{\underset{y=1}{\sum }}I_{\text{initial}-\text{region}}(x,y)}{M\times N},$$ (1)

where $I_{\text{initial}-\text{region}}$ (x,y) of size M×N pixels has a value of 1 for pixels belonging to the detected initial cell regions and a value of 0 for the background pixels. Based on the training dataset, an AF of 0.1 was selected as a threshold for the algorithm to decide if an image is less dense (<0.1) or moderately (≥0.1) dense. In case an image has regions of both low and moderate density, the algorithm would estimate it to be less or moderately dense depending on the predominant region present. An image with a larger region of moderate density is more likely to have an increased AF and be handled as moderately dense. The less-dense cells present would easily be detected as the thresholds for such images were set to facilitate complex cell segmentation. It should be noted that the thresholds used for moderately dense images would not be ideal for images with only less-dense cells as it might lead to a greater number of false detections. On the other hand, regions of moderate density in images classified as less dense would likely be a small cluster of cells. The low-density images have optimal thresholds for detecting markers in such clusters to separate individual cells.

### 2.4 Candidate Cell Region Detection

**Fig. 3:**
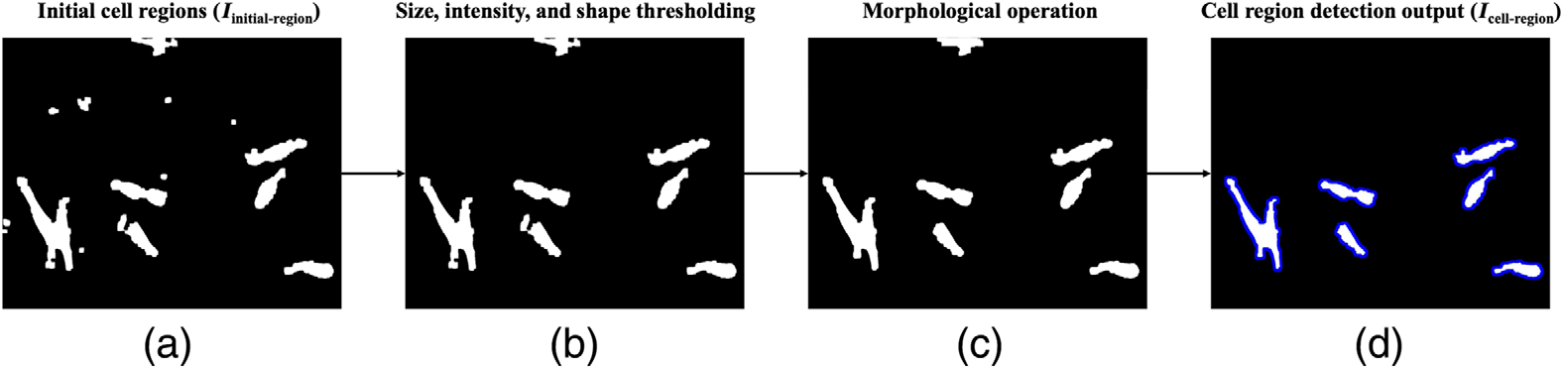
Illustration of the steps involved in identifying candidate cell regions. (a) $I_{\text{initial}-\text{region}}$ obtained after edge detection and morphological operations are used as the input. (b) Thresholding using size, intensity, and shape criteria removes detected objects that are not cells. (c) Opening and erosion optimize the shape of cell areas. (d) Clearing the image border removes incomplete cells resulting in the final candidate cell regions. (Borders of cell regions are highlighted in blue.)

Cell region detection is conducted as a semantic segmentation^41^ step to identify pixels belonging to cells as defined in the truth, and hence, regions detected may contain more than one cell. Figure 3 shows the process of candidate cell region detection. For less dense images, $I_{\text{initial}-\text{region}}$ is used directly to define regions with potential cell objects. Preprocessing is designed to detect objects with high sensitivity [Fig. 3(a)]; therefore, each object is evaluated to remove image artifacts and identify candidate cell regions using thresholding [Fig. 3(b)] and morphological operations [Fig. 3(c)]. For size thresholding, the detected object is removed if the area is less than a threshold value determined by the minimum, maximum, mean, or the standard deviation of the area of all foreground objects in the image. This adaptive approach ensures that the threshold values are not overly biased toward the training set as it takes relative object sizes in each image into account to understand if it is likely to be a cell or not. Similarly, the intensity-based thresholds are calculated using the maximum and minimum intensity values inside that object obtained from the pixel positions in $I_{\text{preprocessed}}$. Objects labeled as cells in the training set contain bright pixels in the cytoplasm and/or dark pixels inside the nucleus after contrast adjustment. Lack of both indicates that the object is not a cell as it has a relatively uniform intensity range similar to substrate. For shape thresholding, circularity and ellipticity features of the object are calculated. From the training data, circularity (mean ± std. dev.) of MSCs and phase imaging artifacts have been found to be 0.43±0.18 and 0.82±0.05, respectively, where 1 represents a perfect circle. Ellipticity is measured as

$$\text{Ellipticity}=\frac{M1-M2}{M1},$$ (2)

where M1 and M2 are the major and minor axis lengths, respectively, of an ellipse having the same normalized second central moment (variance) as the object. Ellipticity (mean ± std. dev.) of MSCs and very thin artifacts such as fibers or strands have been found to be 0.46±0.15 and 0.79±0.04, respectively, where 1 represents a line segment and 0 represents a circle. Since MSCs are not as circular or elliptical as the artifacts, objects with high circularity and ellipticity are removed from the detected cell areas. Morphological operations such as opening and erosion are applied after thresholding to refine boundaries. Finally, objects with pixels connected to the image border are removed to avoid analysis of truncated cells. As shown in Fig. 3(d), these steps yield the final image ($I_{\text{cell}-\text{region}}$) with detected candidate cell regions corresponding to regions of interest (ROI).

For moderately dense images, the edge detection step is performed with a reduced sensitivity threshold of 0.5 to cover all cell edges. Dilation and closing are performed with different structuring element sizes to get new potential cell regions. The same thresholding methods as less dense images are carried out on these objects with different threshold values to keep them relevant for images with more objects. Thresholding is also repeated more times compared to low cell density processing as a lower edge detection threshold can cause more false detections. Thresholding is followed by morphological opening, closing, and border clearing to obtain the $I_{\text{cell}-\text{region}}$ for images having moderate cell density.

### 2.5 Candidate Marker Detection

**Fig. 4:**
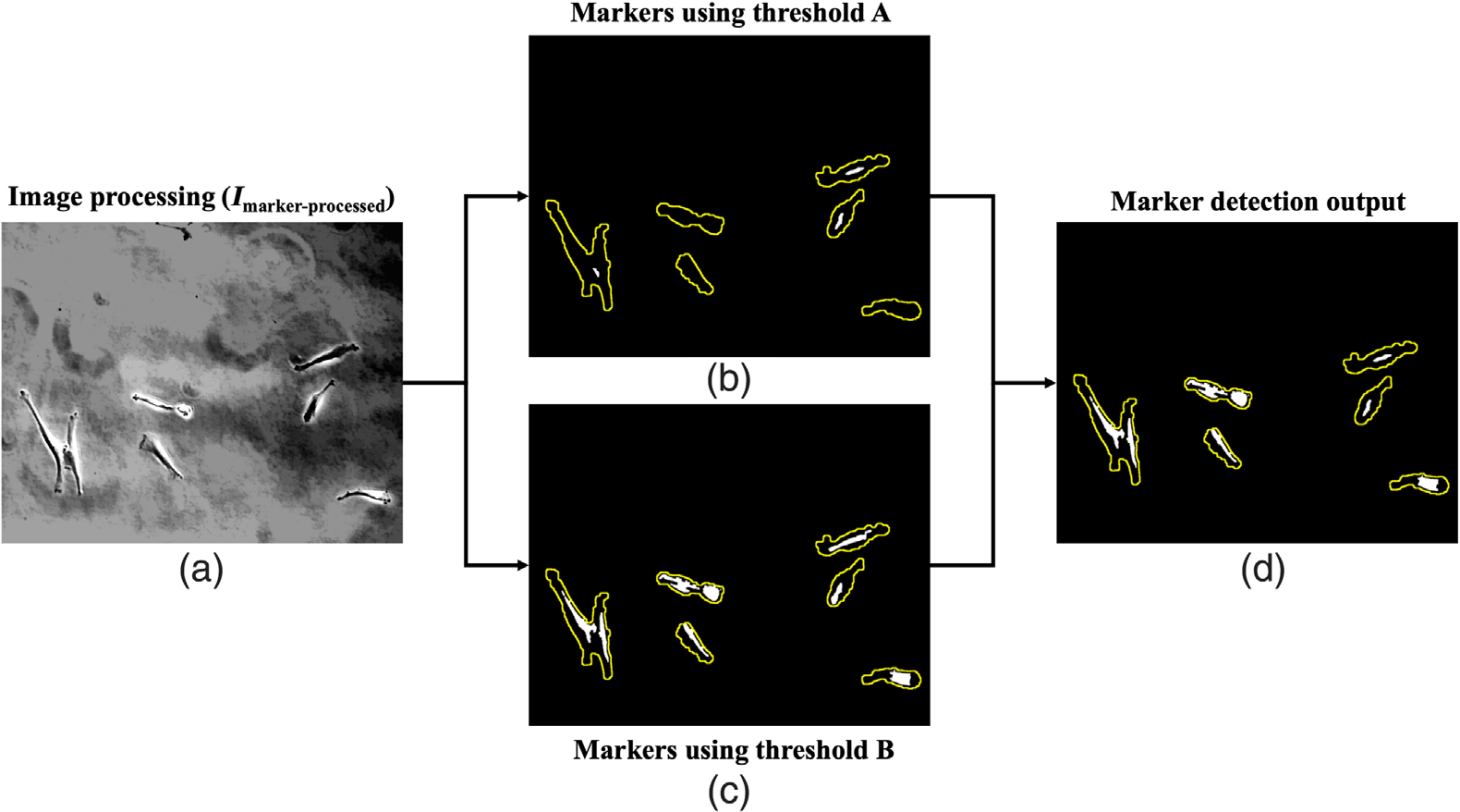
Pipeline for segmentation of markers inside the RoIs (shown in yellow) from $I_{\text{cell}-\text{region}}$. (a) $I_{\text{preprocessed}}$ is processed to get an image ($I_{\text{marker}-\text{processed}}$) suitable for detecting markers. (b) Markers are obtained from $I_{\text{marker}-\text{processed}}$ using H-minima transform with higher threshold A. (c) Markers are obtained from $I_{\text{marker}-\text{processed}}$ using H-minima transform with lower threshold B. (d) Markers from both the thresholds are merged for ROIs satisfying the perimeter threshold criterion and also for ROIs with zero markers from threshold A.

In phase-contrast micrographs of cells, intensity is brightest at the cell boundaries where the phase shift is maximum due to the optical path difference (refractive index and thickness) between cells and substrate, and is darker within the cell due to relative uniformity within the cell.^42^ The darkest regions inside cells are taken as candidate cell markers to identify individual cells and to segment cells in clusters since each cell contains one prominent regional minimum. The image $I_{\text{preprocessed}}$ is further processed using Gaussian filtering and median filtering to remove unnecessary noise and false local minima that do not belong to the marker. Then, contrast-limited adaptive histogram equalization improves the contrast of the regional minimum. Finally, morphological reconstruction is performed using the histogram equalized image along with $I_{\text{cell}-\text{region}}$ as the mask to obtain $I_{\text{marker}-\text{processed}}$, shown in Fig. 4(a).

Markers are segmented using two different threshold values (A and B) for H-minima transform.^43^^–^^45^ Used independently, the higher threshold value of A results in under-detection of markers, and the lower threshold value of B results in false positives (FPs); therefore, the two are combined. First, a very high value is used as threshold A for H-minima transform to localize potential markers inside the ROIs from $I_{\text{cell}-\text{region}}$. Morphological opening and binary area opening are done to remove objects that aren’t the regional minimum [Fig. 4(b)]. Then, minima are obtained with a lower threshold, value B. These minima outputs are dilated and closed to get the candidate markers [Fig. 4(c)]. In the case of zero markers from threshold A in any ROI, markers detected using B are added to that region. Markers from both the thresholds are also merged for potential cluster ROIs using perimeter as a criterion [Fig. 4(d)].

Over-detection error is managed for cell regions with more than one marker using distance thresholding along with area thresholding and morphological operations. The Euclidean distance between the centroid of the markers within a cell region is computed, and if the distance is too small, the regional maximum is over-segmented. Thus, one of the two close markers with the smaller area is removed. Finally, dilation and erosion are performed to get rid of over-segmentations.

### 2.6 Cell Segmentation and Validation

**Fig. 5:**
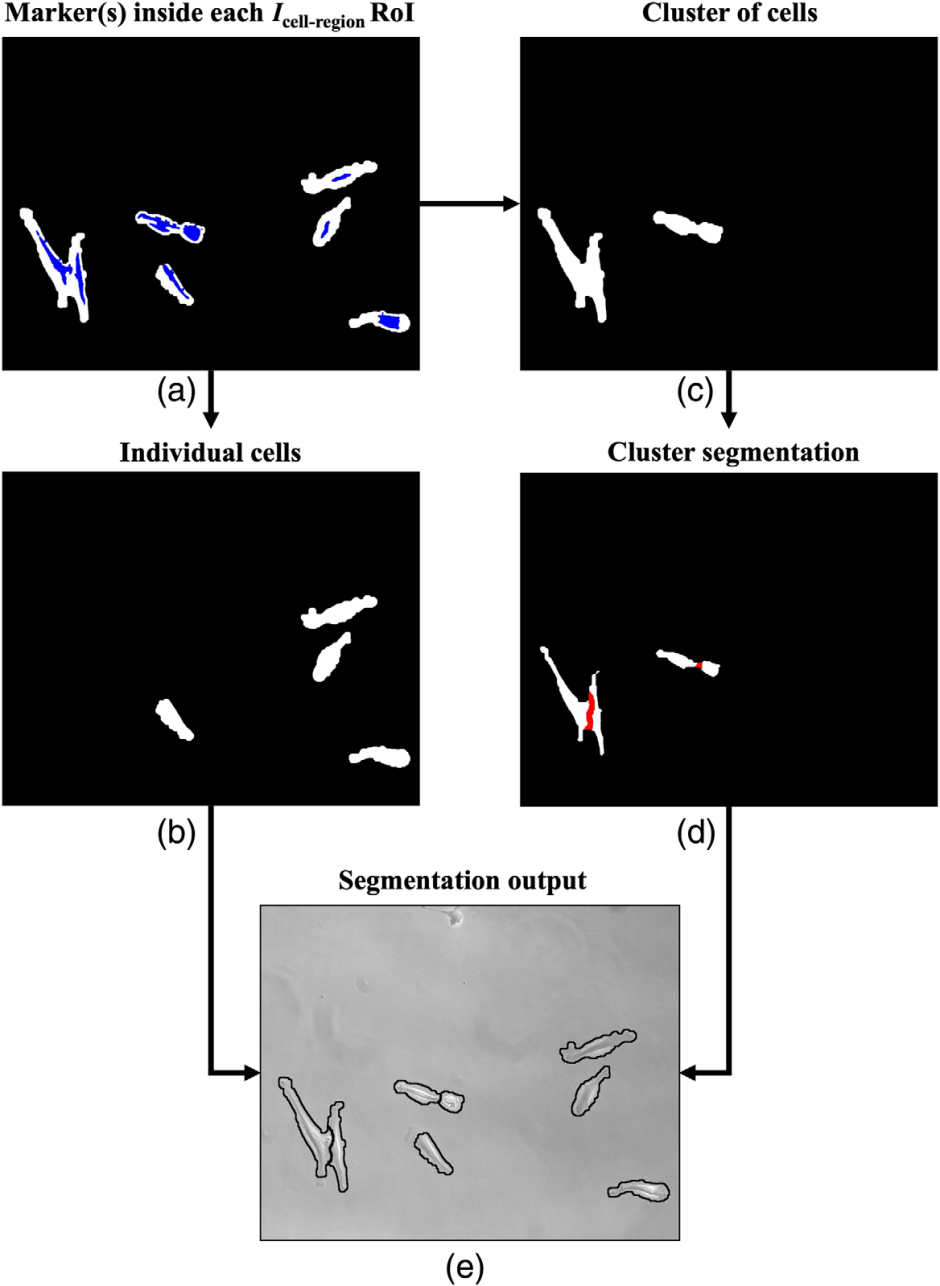
Representation of the cell segmentation steps. (a) $I_{\text{cell}-\text{region}}$ is integrated with the results from marker detection (Markers shown in blue). (b) ROIs with single markers are identified as individual cells, and (c) ROIs with more than one marker are identified as clusters of cells. (d) Marker-controlled watershed is carried out to segment individual cells within clusters (watershed ridge lines shown in red). (e) Results from the steps (b) and (d) are combined to get the segmentation output.

ROIs and markers are combined as shown in Fig. 5(a) for the final instance segmentation^41^ step to detect and delineate each cell in the image. A region with no marker is not considered a cell, only one marker is labeled as a single cell [Fig. 5(b)], and a region with more than one marker is treated as a cluster of cells [Fig. 5(c)]. The marker count within each cluster indicates the number of cells in the cluster. Each cluster region is segmented into individual cells [Fig. 5(d)] using marker-controlled watershed, which overcomes the limitations of standard watershed technique, such as over and under-segmentation, using markers.^46^^,^^47^

The algorithm’s performance for cell detection and segmentation was validated using the expert-defined truth. For cell detection, the true positives (TP) are given by the number of individual cells and cells inside clusters that are correctly detected by the algorithm. The false negatives (FNs) are of two types, namely, missed detections (FN1), where individual cells are not detected, and under-detections (FN2), where the number of detected cells within a cluster is less than the actual number. Similarly, the two types of FPs are false detections (FP1) and over-detections (FP2). FP1 are debris or image artifacts that are erroneously detected as individual cell objects, and FP2 is the number of extra objects detected within a cluster by the algorithm. Sensitivity (S) and precision (P) were computed using

$$S=\frac{TP}{TP+FN1+FN2},$$ (3)

$$P=\frac{TP}{TP+FP1+FP2},$$ (4)

to estimate how well the algorithm is able to detect MSCs. For segmentation, Sorensen–Dice similarity coefficient (DICE)^48^ was used as the metric via

$$\mathrm{DICE}=\frac{2\times |A\cap B|}{\left|A|+|B\right|},$$ (5)

to analyze the agreement between algorithm output (A) and manual outlining (B) for cell boundaries. The DICE score ranges from 0 to 1, with 1 signifying complete overlap between A and B.

The presented algorithm was also assessed by comparing its cell detection and segmentation results with the conventional U-Net,^49^ a deep learning technique for biomedical image segmentation. The U-Net architecture was modeled based on the standard implementation proposed by Ronneberger et al. The conventional U-Net was trained and tested on the same datasets to avoid any bias. Sensitivity, precision, and DICE evaluation metrics were used to compare performance of the algorithm reported here to the standard U-Net.

### 2.7 Feature Extraction

FP objects (FP1 and FP2) and all cells belonging to under-detected (FN2) clusters were excluded from the dataset to avoid training or validating classification models using wrong objects. The final dataset used for feature extraction and classification is detailed in Table 2.

**Table 2:** Mesenchymal stem cell culture dataset used for feature extraction and classification.

	Culture day	No. of cells	No. of RS cells	No. of SR cells
Training	2	167	117	50
(Culture 1 + culture 2)	4	296	182	114
Independent testing	2	49	37	12
(Culture 3)	4	91	54	37

Human engineered descriptors of the correctly detected cells were automatically extracted for the task of classifying each cell into RS or SR phenotype. A total of 30 features, consisting of a combination of size, shape, and first-order and second-order statistical texture measures, were computed.^50^^–^^52^ The names of the features are listed in Table 3. The morphometric features were extracted to distinguish spindle-shaped RS cells from flattened SR cells, whereas texture-based features were extracted to use spatial distribution of intensity for differentiating between SR cells that are flattened and RS cells that have a more prominent phase-contrast halo around their cell body.^16^^,^^40^ Each first-order feature was calculated for segmented cell regions in $I_{\text{gray}}$, $I_{\text{preprocessed}}$, and $I_{\text{reconstructed}}$ giving three measures for each first-order feature. The second-order features were measured for the gray-level co-occurrence matrix (GLCM) of $I_{\text{gray}}$, $I_{\text{preprocessed}}$, and $I_{\text{reconstructed}}$. Also, each GLCM feature was computed in 24 different orientations yielding 72 measures for each second-order feature. Only one measure out of the three and 72 first and second-order feature measures, respectively, was selected by finding the measure with the highest area under the curve (AUC) of the receiver operating characteristic (ROC) curve^53^^,^^54^ for distinguishing between RS and SR cells.

**Table 3:** Human-engineered features extracted for the segmented MSCs.

Feature type	Feature names
Size	Area, perimeter, minor axis, major axis, width, height
Shape	Elongation, compactness, circularity, ellipticity, solidity, extent
First-order features	Standard deviation, variance, intensity profile, skewness,
	mean intensity, balance, kurtosis, median, mode
Second-order features	Correlation, inertia, cluster prominence, energy, entropy,
	cluster shade, maximum probability, dissimilarity, homogeneity

The 30 features were sorted in descending order of their AUC value before computing the correlation matrix to ensure that features with higher AUCs were retained. Features with correlation >0.8 were removed to reduce redundancy and optimize the computation for the classifier.

### 2.8 Cell Classification and Validation

The selected features were used to train linear and non-linear classifiers to find the most suitable model for our application. Features were transformed to have zero mean and unit variance before training, and the validation/test data were scaled using the training parameters. As seen in Table 1, the dataset is imbalanced with RS cells being more prevalent than the SR cells. To avoid poor classification of SR cells due to its low prevalence, synthetic minority oversampling technique^55^ was used during training.

Linear kernel support vector machine (LSVM), radial-basis kernel support vector machine (RSVM), linear discriminant analysis (LDA), K-nearest neighbor (KNN), and logistic regression (LR) models were trained to classify MSCs as RS or SR phenotype. The models were trained using features from both day 2 and day 4 together, and day 2 and day 4 individually. As the feature correlation and relevance for data from day 2, day 4, and combination of day 2 and 4 would be different, features were selected for each of them individually. It was observed that the classifier could learn better from features of cells whose images were acquired on the same day rather than the combination of features of cells from two different days. AUC values were obtained using ROCKIT software,^56^ and all the models were compared using the average AUC value from five-fold cross-validation.^57^ Further, ensemble classifiers based on soft-voting method were also trained with the top two classifiers for day 2 and day 4 separately.^58^ Finally, the performance of the selected models for the independent test dataset was evaluated using AUC, sensitivity, and specificity. Sensitivity and specificity were determined by selecting a threshold that minimizes $(1-\text{sensitivity}{)}^{2}+{\left(1-\text{specificity}\right)}^{2}$.^58^

## 3 Results

### 3.1 Cell Detection and Segmentation

The algorithm’s ability to accurately locate MSCs was evaluated using sensitivity and precision. Table 4 shows the breakdown of correctly detected cells and undetected cells for RS and SR cells in the training and independent testing sets. FNs are further defined as missed detections (FN1) or under-detections (FN2) as described in Sec. 2.6. The algorithm correctly detected cells with sensitivity >0.95 for both RS and SR cell phenotypes in the training set. A sensitivity over 0.8 in the independent testing set indicates the robustness of the algorithm.

**Table 4:** Cell detection sensitivity of the algorithm for training and independent testing.

	Cell phenotype	No. of cells	Correct detections (TP)	Missed detections (FN1)	Under detections (FN2)	Sensitivity (S)
Training	All	472	466	3	3	0.987
	RS	307	302	2	3	0.984
	SR	165	164	1	0	0.994
Independent testing	All	186	157	13	16	0.844
	RS	121	102	8	11	0.843
	SR	65	55	5	5	0.846

Precision is the fraction of objects detected by the algorithm that were identified as cells in the ground truth labeling of the dataset. Table 5 shows the number of incorrectly identified objects that are either false detections (FP1) or over-detections (FP2). The algorithm’s precision for cultures used in training was above 0.95. A precision >0.85 in the independent testing set demonstrates the generalizability of the algorithm to detect cells with a low FP rate.

**Table 5:** Cell detection precision of the algorithm for training and independent testing.

	Correct detections (TP)	False detections (FP1)	Over detections (FP2)	Precision (P)
Training	466	5	8	0.973
Independent testing	157	13	13	0.858

The DICE metric was used to evaluate the overlap between the algorithm and the ground truth segmentation of MSCs. DICE scores (mean ± std. dev.) for the training and testing dataset are summarized in Table 6 for all cells outlined in the truth and only for the cells that were correctly detected. The algorithm segmented over 85% and 80% of the manually defined cell areas for training and testing, respectively, regardless of cell phenotype.

**Table 6:** Cell segmentation DICE score of the algorithm for training and independent testing.

	Cell phenotype	DICE score (mean ± std. dev.)	
		All cells (TP + FN1 + FN2)	Correctly detected cells (TP)
Training	All	0.875±0.092	0.881±0.067
	RS	0.878±0.089	0.884±0.061
	SR	0.870±0.097	0.875±0.077
Independent testing	All	0.803±0.218	0.869±0.082
	RS	0.814±0.203	0.871±0.080
	SR	0.783±0.245	0.866±0.085

The performance of the algorithm for cell detection and segmentation were analyzed per each cell in Tables 3–5. Sensitivity, precision, and DICE metrics of the algorithm were further examined per image from all cultures and days. The mean ± std. dev of these metrics for the training and testing images are given in Table 7, and it was confirmed that the algorithm could detect and segment cells consistently for each image.

**Table 7:** Cell detection and segmentation performance of the algorithm per image from training and testing datasets. Mean and standard deviations are calculated for all cells over all images.

	No. of images	Cell detection sensitivity (mean ± std. dev.)	Cell detection precision (mean ± std. dev.)	Cell segmentation DICE score (mean ± std. dev.)
Training	71	0.991±0.036	0.967±0.107	0.896±0.050
Independent testing	36	0.837±0.207	0.861±0.217	0.849±0.106

**Fig. 6:**
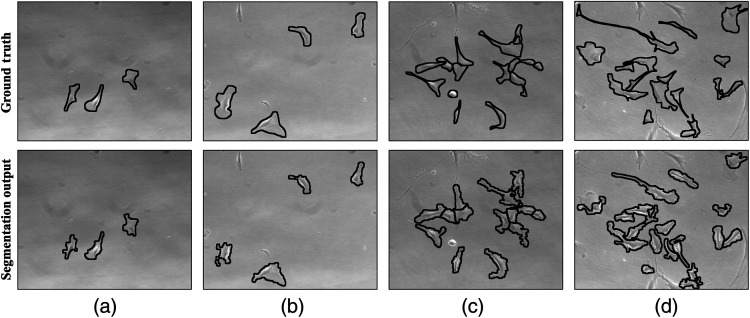
Sample images of MSCs from the independent test dataset comparing the ground truth cell outlines (top row) with the segmentation results of the algorithm (bottom row). Columns (a) and (b) are examples of low-density images whose AF values were estimated as 0.04 and 0.08, by the algorithm, respectively. Similarly, examples of moderate density images with AF values of 0.15 and 0.21 are shown in column (c) and (d), respectively. The ground truth images also illustrate that the truncated cells on image borders are not considered for analysis.

Figure 6 shows the ground truth along with algorithm’s segmentation results for two images of both low and moderate density from the test dataset. The difference in performance of the algorithm to localize cells in low and moderately dense images was also reviewed as it was trained with different parameters for these two levels of cell densities. Welch’s t-test at 95% confidence level failed to show statistical difference in the values of sensitivity (p=0.375), precision (p=0.191), and DICE (p=0.289) for low and moderately dense cell images.

The cell detection and segmentation results of the algorithm are compared against the conventional U-Net in Table 8 for both training and testing data. The presented algorithm outperforms the conventional method in all of the evaluation metrics for this task. The low precision of the U-Net was determined to be due to detection of several image artifacts as cells. The algorithm was able to overcome such FP detections through various morphological thresholding steps. Moreover, although the U-Net model used weighted maps to learn the small separation borders, it could not separate cells in clusters as effectively as the marker-controlled watershed technique used in the presented pipeline. Additional work would be needed to modify and optimize U-Net’s architecture for it to be able to localize MSCs more accurately. Thus, it was statically demonstrated using paired two-sample t-tests that the algorithm could detect and segment cells significantly better than the state-of-the-art method.

**Table 8:** Comparison of algorithm’s cell detection and segmentation performance with conventional U-Net.

		Sensitivity (S)	Precision (P)	Sensitivity per image (mean ± std. dev.)	Precision per image (mean ± std. dev.)	DICE score per image (mean ± std. dev.)
Training	Conventional U-Net	0.765	0.653	0.817±0.173	0.692±0.213	0.841±0.056
	Presented algorithm	0.987	0.973	0.991±0.036	0.967±0.107	0.896±0.050
Independent testing	Conventional U-Net	0.747	0.556	0.772±0.216	0.536±0.210	0.804±0.050
	Presented algorithm	0.844	0.858	0.837±0.207	0.861±0.217	0.849±0.106
	p-value	N/A	N/A	0.025	5.259e – 10	0.008

### 3.2 Cell Phenotype Classification

**Fig. 7:**
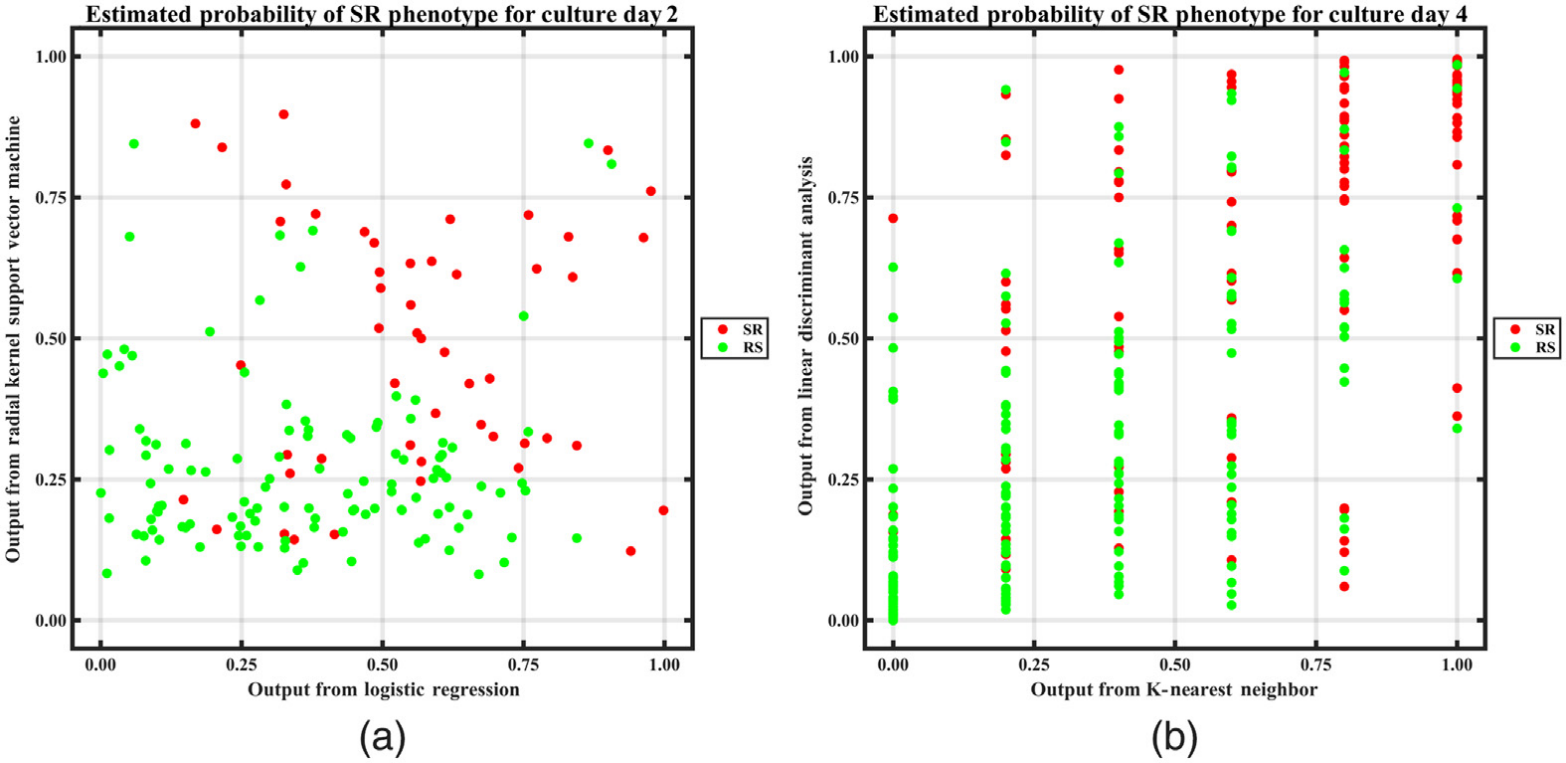
Diagonal classifier agreement plots between (a) logistic regression classifier (x axis) and radial kernel support vector machine (y axis) for day 2 culture and (b) KNN (x axis) and linear discriminant analysis (y axis) for day 4 culture. Each point represents a cell whose phenotype is predicted as RS (green) or SR (red) by each classifier.

As mentioned previously, only correctly detected individual cells and cells in clusters with correct cell count were used for training and testing the machine learning models for the task of classifying each cell phenotype as RS or SR. LSVM, RSVM, LDA, KNN, and LR were trained and compared using the AUC metric from five-fold cross-validation. These five classifiers trained using object features from both day 2 and day 4 cultures were validated by testing their performance in cross-validation for a combination of cells from “day 2 + day 4,” and day 2 and day 4 independently. It was observed that day 2 + day 4 models did not perform as well for day 2 as they did for day 4 (Table 9). This may be due to models’ bias toward a higher prevalence of day 4 MSC features. Training the classifiers with features from day 2 and day 4 individually improved their performance for both days (Table 9). The outputs from the top two classifiers (RSVM and LR for day 2; LDA and KNN for day 4) based on cross-validation AUC were combined for ensemble classification. Figure 7 shows the classifier agreement plot between the models used for ensemble classification. The x axis in Fig. 7(b) is not continuous due to the discrete probability distribution of the KNN classifier. The disagreement between the top 2 classifiers for each day may be because they learn differently from the same features. “RSVM + LR” and “LDA + KNN” models being a combination of linear and non-linear approaches are able to harness the potential of both the models to make more accurate predictions. This likely explains why fusion classifiers perform better than the individual classifiers during five-fold cross-validation, and hence, they are selected for the image analysis pipeline.

**Table 9:** Five-fold cross-validation to compare performance of models for distinguishing between RS and SR cells. AUC values are in bold for Day 2 and Day 4 classifiers that perform best during cross-validation.

	Five-fold cross-validation AUC ± S.E. (culture 1 + culture 2)				
Training fold	Day 2 + Day 4			Day 2	Day 4
Testing fold	Day 2 + Day 4	Day 2	Day 4	Day 2	Day 4
LSVM	0.734±0.012	0.673±0.033	0.751±0.022	0.719±0.044	0.810±0.026
RSVM	0.752±0.012	0.682±0.008	0.775±0.025	0.736±0.057	0.799±0.024
LDA	0.733±0.017	0.679±0.035	0.748±0.033	0.698±0.044	0.833±0.016
KNN	0.725±0.014	0.677±0.035	0.738±0.018	0.719±0.059	0.828±0.022
LR	0.747±0.014	0.696±0.030	0.765±0.027	0.731±0.043	0.821±0.021
RSVM + LR	N/A	N/A	N/A	0.757±0.041	0.844±0.038
LDA + KNN	N/A	N/A	N/A	0.745±0.064	0.863±0.030

**Fig. 8:**
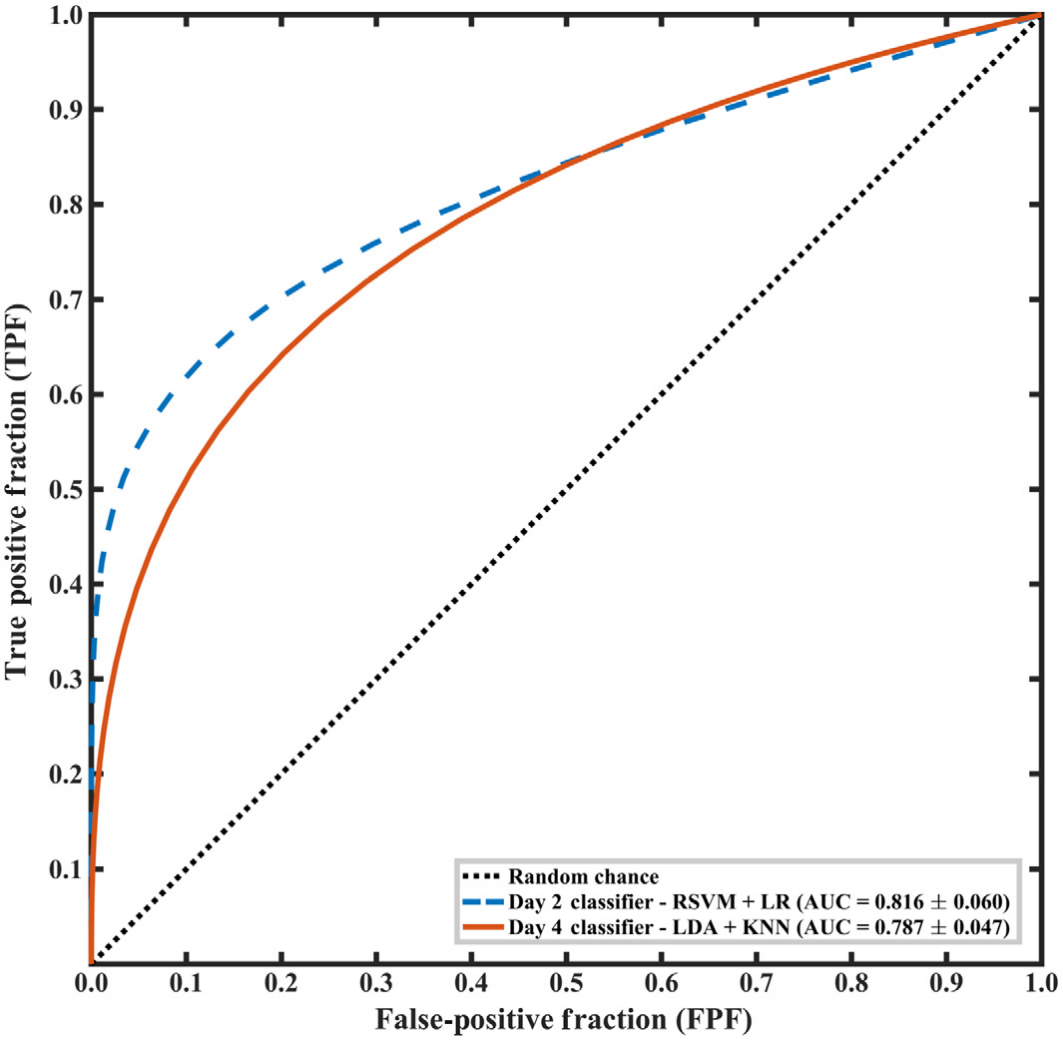
Fitted binormal ROC curves illustrating the performances of day 2 and day 4 ensemble classifiers for the task of distinguishing between RS and SR cells during independent testing. The dashed blue line represents the RSVM + LR day 2 classifier and the solid orange line represents the LDA + KNN day 4 classifier.

The selected ensemble classifiers for day 2 and day 4 are further evaluated using the independent test dataset. The algorithm was able to correctly identify cell phenotypes with an AUC of 0.82 for day 2 and 0.79 for day 4. The classification models for both days have been statistically proven to perform better than random chance using the 95% confidence interval for AUCs given in Table 10. These inferences were not corrected for multiple comparisons as only a single statistical test was performed for each day. The fitted ROC curves obtained for the day 2 and day 4 ensemble classifiers are shown in Fig. 8. The algorithm achieves a sensitivity and specificity of over 0.75 for both days, further demonstrating its effectiveness in classifying MSCs based on their phenotype.

**Table 10:** Performance of the algorithm for classifying MSCs as RS or SR for independent testing.

Culture day	Model	AUC ± S.E. (95% CI)	Sensitivity	Specificity
2	RSVM + LR	0.816±0.060 (0.769, 0.886)	0.789	0.887
4	LDA + KNN	0.787±0.047 (0.716, 0.851)	0.796	0.757

### 3.3 Generalizability of Phenotype Classification

The cells in the training and test dataset were also labeled by a group of 20 individuals with varied level of expertise in working with MSCs. The group comprised of nine people with one to three years of experience, six people with four to nine years of experience, and five people with ten or more years of experience. This analysis was performed to study the generalizability of the developed algorithm as phenotype assessment of MSCs is highly subjective. Each cell was classified as either RS (0) or SR (1) by all 20 individuals, and its average phenotype score (APS) was computed. Based on the range of average scores, the cells were split into categories such as highly RS, moderately RS, uncertain, moderately SR, and highly SR (Table 11). The confidence of the group in identifying a cell as either RS or SR was represented by these five categories; where the highly RS/SR categories meant highest confidence in labeling that cell and uncertain category meant that the phenotype of that cell was almost indeterminate.

**Table 11:** Classification sensitivity of the algorithm for the five cell phenotype categories.

	Culture day	No. of cells correctly classified/no. of cells as per truth (sensitivity)				
		Highly RS APS≤0.15	Moderately RS 0.15<APS<0.40	Uncertain 0.40≤APS≤0.60	Moderately SR 0.60<APS<0.85	Highly SR APS≥0.85
Training	2	40/40 (1.000)	36/40 (0.900)	29/38 (0.763)	29/33 (0.879)	16/16 (1.000)
	4	10/10 (1.000)	9/11 (0.818)	5/12 (0.417)	11/13 (0.846)	3/3 (1.000)
Independent testing	2	72/74 (0.973)	66/77 (0.857)	26/46 (0.565)	57/65 (0.877)	31/34 (0.912)
	4	15/16 (0.937)	17/21 (0.809)	7/19 (0.368)	20/23 (0.869)	12/12 (1.000)

The output of the ensemble classifiers for day 2 and day 4 from training and testing set was studied to understand which category of the cells were most wrongly predicted by the algorithm. It was noticed that the algorithm had the highest classification sensitivity (>0.9) for highly RS and highly SR categories and the least sensitivity (as low as 0.4) for the uncertain category (Table 11). This enabled us to understand that while our image analysis pipeline has the potential to predict the cell phenotype with a sensitivity very close to human interpretation, it did exhibit a similar weakness to trained humans in that it had lower sensitivity in categorizing marginal morphological characteristics. If these limitations are addressed in future iterations of the algorithm, this approach has the potential to out-perform human observers with decades of experience.

## 4 Discussion and Conclusions

The image analysis method reported here is capable of segmentation and classification of MSCs based on their morphological phenotype. Segmentation results provide a cell count per image, cell density ($\text{cells}/{\mathrm{cm}}^{2}$), and percent confluency that indicate cell proliferation over the time course of the culture. Classification of segmented cells yields a count of undesirable SR cells (quiescent) and the ratio of these non-viable cells to viable RS cells (high potency) that serves as a vital indicator of culture quality. The promising sensitivity, precision, and DICE score for MSC localization in phase-contrast micrographs suggest that automated quantitative evaluation can be seamlessly integrated into the current cell culture workflow.

That being said, it is crucial to note that an overall evaluation of the developed image analysis approach has not been included in this study. It was only feasible to assess each individual stage of the algorithm separately using the available dataset and an additional independent dataset would be needed for overall validation of the entire system. The cell detection and segmentation stages are greatly influenced by the intensity distribution, contrast, and clarity of the input phase-contrast micrographs. The classification stage in turn is dependent on the segmentation output. Upon resolution of these challenges, classifiers could be trained for more robust prediction by expanding the dataset and accounting for cells whose phenotype is uncertain. These challenges faced by different stages of the pipeline are discussed in detail subsequently.

The ability of the algorithm to detect and segment cells is greatly dependent upon the quality of the acquired images. In phase-contrast micrographs, high contrast is crucial to distinguish cells from the substrate. The majority of detection errors during training and testing were primarily due to poor image contrast and blur in some image regions. Fluorescence microscopy of labeled cells would provide higher contrast images and easier segmentation; however, phase-contrast microscopy is the standard technique for noninvasive evaluation of live cells. The low rate of FP and FN detections has minimal impact on the overall quality assessment as long as a sufficient number of images are acquired to capture a population of correctly segmented cells.

The classification results were studied to understand factors that affect the performance of proposed models. In this study, classifiers were trained using the truth defined by a biologist with 15+ years of experience working with MSCs. The majority of MSCs incorrectly classified were identified as cells undergoing differentiation from RS to SR phenotype. Additionally, phenotype labels were obtained for the entire dataset from 20 trained individuals with varying levels of expertise in culturing MSCs to assess the subjectivity of human classification and the generalizability of the algorithm. This labeling enabled an analysis of the impact of ambiguity in morphology of differentiating cells on visual inspection and classification. The trained individuals had minimal agreement about cell phenotype during differentiation, demonstrating the uncertainty in classification of cells in this transition both for human interpretation and the algorithm. The existing binary classifier could be trained as a multiclass problem with the task of distinguishing between RS, SR, and these indeterminate cells. Identifying cells with indeterminate phenotype would decrease the number of false classifications and increase certainty in prediction of RS and SR phenotypes. However, more data would be necessary to train the machine learning model to predict indeterminate class effectively. An alternative would be to calibrate the binary classifier that differentiates RS from SR cells for correlating its probability output with confidence in a cell’s phenotype. The future work would be to fine-tune the current approach by adopting the above-mentioned machine learning methods to improve classification robustness.

Classification performance was only validated for cells that were correctly detected and were not evaluated for FP objects. Since this technique would be implemented in real-time as a pipeline where every segmented object would be classified, it is necessary to evaluate the effect of classification of incorrectly detected objects. Though not in the scope of this article, it may also be interesting to analyze if the algorithm tends to classify FP objects as RS or SR cells. Images from three different cell cultures have been used for the dataset here. This algorithm can be refined and tested using a broader set of images from MSC cultures generated in other laboratories and obtained with different phase-contrast microscopes. The phenotype of cells from day 2 and day 4 is predicted using two different classifiers. This was done because a day 2 + day 4 model was biased against cells from day 2 due to limited data as compared to day 4 when more cells are present. Additional data from more cultures may enable sufficient features for day 2 cells to train a common classifier with greater prediction capacity, removing the need for two separate classifiers. Apart from this, a larger dataset will not only increase data variability for algorithm training but also enable characterization of the capability to predict quality of cultures during practical application. The algorithm’s performance can also be compared to standard culture evaluation assays, many of which are time consuming and labor intensive, to evaluate quantitative phenotypic analysis as a measure of replication potential for MSC cultures. These considerations do not include the considerable amount of time, effort, and expense associated with training expert observers.

The focus of the research presented in this paper has been to prove the applicability of image-based analysis for non-invasive and objective determination of MSC phenotype in low and moderately dense cultures. The algorithm is not expected to perform well for highly dense cell images where there is a lot of cell overlap making even visual investigation complicated. Quantitative evaluation of the earlier stages of culture is more critical for monitoring the health of the culture. MSC cultures are typically harvested or passaged prior to high confluency. This methodology has the potential to be extended to:

• Forecast the percentage of non-functional MSCs in a culture for a future time based on the ratio computed for the present and past time points.
• Quantitatively represent the influence of change in culture protocol over the population of putatively efficacious and non-functional MSCs in a culture.
• Monitor the ratio of non-functional to putatively efficacious MSCs as a function of confluency and cell density in addition to time.
• Estimate efficacy of other stem cell cultures for their prospective use in cytotherapies using image-based morphological analysis.

In summary, we have shown that the presented analysis can segment and classify MSCs based on their morphological phenotype to quantify the viability of monolayer cultures. As this computational pipeline is completely non-invasive, it enables continuous monitoring of culture conditions to enhance reproducibility. It is anticipated that this algorithm will facilitate biologists and cell manufacturers to draw conclusions about the functionality of recovered MSCs. The proposed solution with automated imaging leads to rapid, quantifiable, and standardized MSC quality control processes. It could be incorporated into high-volume stem cell manufacturing to pave the way for efficient cell therapies to treat chronic diseases.
